# NGS Custom Panel Implementation in Patients with Non-Syndromic Autism Spectrum Disorders in the Clinical Routine of a Tertiary Hospital

**DOI:** 10.3390/genes14112091

**Published:** 2023-11-17

**Authors:** Ana Karen Sandoval-Talamantes, Jair Antonio Tenorio-Castaño, Fernando Santos-Simarro, Carmen Adán, María Fernández-Elvira, Laura García-Fernández, Yolanda Muñoz, Pablo Lapunzina, Julián Nevado

**Affiliations:** 1 INGEMM (Institute of Medical and Molecular Genetics), La Paz University Hospital, IdiPAZ, 28046 Madrid, Spain; dra_talamantes@hotmail.com (A.K.S.-T.); jairantonio.tenorio@gmail.com (J.A.T.-C.); fernando.santos@salud.madrid.org (F.S.-S.); carmen.adan@salud.madrid.org (C.A.); mfelvira@salud.madrid.org (M.F.-E.); lauragfdez@gmail.com (L.G.-F.); ymunozg@salud.madrid.org (Y.M.); plapunzina@gmail.com (P.L.); 2 ITHACA, European Research Network, La Paz University Hospital, 28046 Madrid, Spain; 3 CIBERER (Network for Biomedical Research on Rare Diseases), Carlos III Health Institute (ISCIII), 28046 Madrid, Spain

**Keywords:** autistic spectrum disorder, panel, NGS, tertiary hospital

## Abstract

Autism spectrum disorder (ASD) is a set of neurodevelopmental disorders characterized by deficiencies in communication, social interaction, and repetitive and restrictive behaviors. The discovery of genetic involvement in the etiology of ASD has made this condition a strong candidate for genome-based diagnostic tests. Next-generation sequencing (NGS) is useful for the detection of variants in the sequence of different genes in ASD patients. Herein, we present the implementation of a personalized NGS panel for autism (AutismSeq) for patients with essential ASD over a prospective period of four years in the clinical routine of a tertiary hospital. The cohort is composed of 48 individuals, older than 3 years, who met the DSM-5 (The Diagnostic and Statistical Manual of Mental Disorders) diagnostic criteria for ASD. The NGS customized panel (AutismSeq) turned out to be a tool with good diagnostic efficacy in routine clinical care, where we detected 12 “pathogenic” (including pathogenic, likely pathogenic, and VUS (variant of uncertain significance) possibly pathogenic variations) in 11 individuals, and 11 VUS in 10 individuals, which had previously been negative for chromosomal microarray analysis and other previous genetic studies, such as karyotype, fragile-X, or MLPA/FISH (Multiplex Ligation dependent Probe Amplification/Fluorescence in situ hybridization) analysis. Our results demonstrate the high genetic and clinical heterogeneity of individuals with ASD and the current difficulty of molecular diagnosis. Our study also shows that an NGS-customized panel might be useful for diagnosing patients with essential/primary autism and that it is cost-effective for most genetic laboratories.

## 1. Introduction

Autism Spectrum Disorder (ASD) is a disorder belonging to the generalized group of conditions categorized as neurodevelopmental disorders (NDD). Its main clinical characteristics are deficits in communication, social interaction, and repetitive and restrictive behaviors. In 1997, Lorna Wing proposed the term “autism spectrum disorder” to describe the clinical variability of autism, which includes typical childhood autism, Asperger syndrome, and pervasive developmental disorders not otherwise specified in another category, as described in the International Classification of Diseases (ICD-10.1) and the DSM-5, the fifth edition of the Diagnostic and Statistical Manual of Mental disorders. The diagnosis of ASD is conventionally established in children older than 3 years of age [1,2,3].

ASD occurs more frequently in males, with a ratio of four men for every affected woman, and is the most prevalent childhood neuropsychiatric condition, with a rate of 1.43 per 1000 people [4]. In Spain, 1/156 people (0.64%) have ASD [5]. Some studies estimate a genetic susceptibility in ASD between 40–80% [6]. Currently, in at least 70–75% of patients, the underlying genetic cause remains unknown [7].

The diagnostic causes of autism can be classified as chromosomal (5%), copy number variations (CNVs) (10–20%), monogenic causes (5%), and the rest, associated with metabolic disorders, syndromes of indeterminate etiology, and environmental exposures [8]. Furthermore, ASD can be non-associated to genetic syndromes (primary, essential) or related to more than 100 genetic disorders [9,10]. Among the most frequent are fragile X syndrome (OMIM #300624), tuberous sclerosis (OMIM #191100), Down syndrome (OMIM #190685), and Rett syndrome (OMIM #312750). However, these well-known genetic disorders represent a very small percentage (less than 5%) of the identifiable causes of ASD [11,12].

Currently, due to the potential link of ASD to genetic/genomic anomalies, the use of genomic techniques, including chromosomal microarrays (CMA) and next-generation sequencing (NGS), is highly recommended for its diagnosis [13]. Other techniques that are also used for the detection of genetic/genomic changes in ASD patients are karyotyping, fluorescence in situ hybridization (FISH), multiplex ligation-dependent probe amplification (MLPA), and triplet expansion studies for fragile X. The percentage of cases that have an established genetic condition detected by these different molecular techniques ranges from 20% to 25% [8,14,15].

NGS is a sequencing technique that can be approached using three types of designs: (i) Sequencing of selected gene panels (disease panel) that could represent around 0.05% of the genome; (ii) Whole exome sequencing (WES) that analyzes coding regions or exons and represents 1–2% of the genome; (iii) Whole genome sequencing (WGS) that includes intronic regions and intergenic DNA regions [13]. The use of NGS has helped to establish that there are at least 156 to 280 genomic regions that contribute to the development of ASD. Whole exome sequencing over off 900 individuals has estimated the existence of more than 1000 genes contributing to the pathogenesis of this disease. The most frequent genes with variants in their sequence found are *NLGN3*, *NLGN4*, *SHANK2*, *SHANK3*, *NRXN1*, *NRXN3*, *PTCHD1/PTCHD1AS*, *SHANK1*, *DPYD*, *ASTN2*, *DPP6*, *MBD5*, *CDH8*, *CNTNAP*, *SNRPN*, *UBE3A*, *ATP10A*, *GABRB3*, *OCA2*, *APBA2*, *NDNL2*, *TJP1*, *TRPM1*, *KLF*, *CHRNA7*, *SCG5*, *LAT-4*, *SPN*, *MAZ*, *MVP*, *SEZ6L2*, *HIRIP3*, *DOC2A*, *MAPK3*, and *CD2BP2.* It is important to note that most of these genes act on neurotransmission in the central nervous system [16,17]. However, to date, no isolated gene nor locus has been considered as a factor contributing to more than 0.8% of ASD cases in particular cohorts [15,18,19].

Studies using NGS related to ASD in the Spanish population are scarce. Only one study used a custom-designed panel of 44 genes trying to increase the yield in 50 high-functioning ASD patients, where 22 rare heterozygous variants were identified in 21 patients, but only 6 of them (13.6%) were considered pathogenic [20]. Therefore, it is important to describe known genes and to identify new candidate genes through NGS to expand our understanding of the genetic contribution in ASD patients worldwide. Such information would assist in establishing the best implementation for an ASD patient’s individual clinical routine analysis in a tertiary hospital.

## 2. Materials and Methods

Participants were recruited prospectively between 2016 and 2019 from patients receiving routine care at the genetic or neuropediatric clinics of La Paz University Hospital in Madrid, Spain, excluding those with a previously abnormal karyotype, positive fragile-X syndrome study, or suspicion of an identifiable syndromic entity confirmed by other techniques such MLPA or FISH. Patients who had undergone diagnostic evaluation by geneticists or neurologists and showed a genetically validated and identifiable monogenic disease or syndrome, multifactorial cause, exposure to teratogens, or metabolic syndromes were also excluded. Each individual underwent genetic and clinical evaluations of their cognitive abilities and ASD symptoms to ensure non-syndromic ASD phenotypes. Data collection and sampling were performed with prior written informed consent.

**Figure 1:**
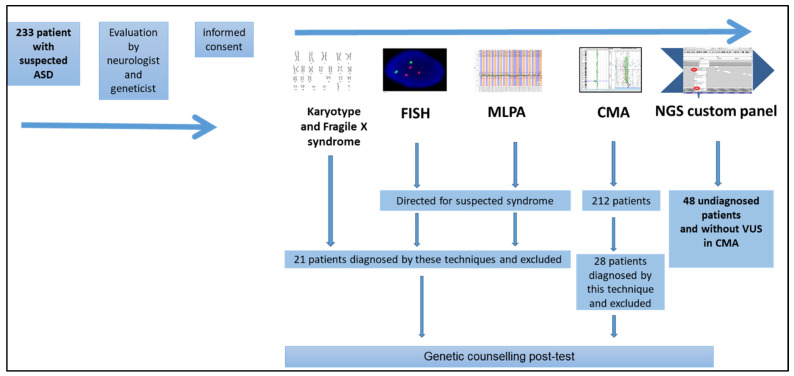
Patient selection algorithm. ASD—Autistic Spectrum Disorder; FISH—fluorescence in situ hybridization; MLPA—Multiplex Ligation-Dependent Probe Amplification; CMA—Chromosomal Microarray Analysis; NGS—Next Generation Sequencing.

Initially, 233 patients aged > 3 years with non-syndromic ASD and DSM-V diagnostic criteria were recruited, but some cases were excluded due to inadequate DNA quality, resulting in a final cohort of 212 individuals. After performing different genetic studies, including CMA, on these patients, we randomly selected 48 individuals (with previous negative results) to perform a personalized NGS panel (Figure 1).

### 2.1. Genetic Studies

#### 2.1.1. Karyotyping and Fluorescence In Situ Hybridization (FISH) Analysis

Karyotyping analyses were conducted on GTG-banded metaphases at an approximate resolution of 550 bands. The analyses followed standard laboratory protocol utilizing Chromosome Kit P (Euroclone; Siziano, PV, Italy). Additionally, FISH analyses were performed using various probes from Kreatech Biotechnology B.V (Amsterdam, The Netherlands) and Vysis Inc. (Downers Grove, IL, USA), according to standard laboratory protocols.

#### 2.1.2. Fragile-X Syndrome Analysis

TP-PCR was utilized to quantify the CGG repeat sequences in the FXS region of the *FMR1* gene. The procedure was performed using a LabGscan FRAXA commercial kit from Diagnostica LongWood (Zaragoza, Spain), following the manufacturer’s instructions. Subsequently, the products were migrated into the 3130 xl ABIPrism genetic autoanalyzer from Thermo-Fischer (Waltham, MA, USA).

#### 2.1.3. Multiplex Ligation-Dependent Probe Amplification (MLPA) Analysis

Different MLPA kits (P036, P070, P245, P373, P064, and P096) were utilized alongside Salsa kits to detect microdeletion/microduplication syndromes. All kits were sourced from MRC-Holland (Amsterdam, The Netherlands). Data analysis was performed following the manufacturer’s protocols using Coffalysser v.9.4, also from MRC-Holland (Amsterdam, The Netherlands).

#### 2.1.4. Chromosomal Microarray (CMA) Analysis

CMA analysis was performed using our custom platform (KaryoArray^®^ v3.0) [21]. The data were analyzed with the CytoGenomics software (v.5.3; Agilent Technologies, Santa Clara, CA, USA) using the default CGH analysis method and interpreted by referencing available databases such as DGV, ClinVar, DECIPHER, and STRING. We previously established the absence of clinically significant CNVs in the 48 samples used in this study [22].

#### 2.1.5. Next Generation Sequencing (NGS) Custom Panel Design

The designed panel (AutismSeq v1.2) includes genes related to both primary autism and/or syndromic autism and was designed by us using the NONACUS tool, based on NONACUS technology (Birmingham, UK) and verified by autism key opinion leaders (KOL), our clinical experience, as well as using curated specific autism databases such as SFARI, AUTISMKB, AUTdb, and GeneCards, among others.

Although we initially started from 523 genes, in this panel, we only included genes with scientific significance associated with ASD. The initial version (v1.1) included 450 genes (the year 2021). Finally, the AutismSeq v1.2, consisting of 311 genes (see Table S1 of Supplemental data), was used. To carry out the validation of the panel, 10 previously diagnosed individuals with known pathogenic variations in different genes were included as controls (see Table S2 of Supplemental data).

#### 2.1.6. Next Generation Sequencing (NGS) Custom Panel Analysis

For the study of 48 patients through NGS (the number of patients for this pilot study has been determined by the NGS kit limitation panel, supporting up to 48 patients per assay), a custom panel (AutismSeq v1.2) was used in individuals who had been previously analyzed by CMA and who did not have any clinically significant CNV (14 of them with benign ones and 34 without previous CNVs) (see ref. [21]). Sequences were captured using NONACUS technology (Birmingham, UK) and subsequently sequenced on an Illumina NovaSeq 6000 platform (Illumina, San Diego, CA, USA) at IMEGEN (new Health in Code, Madrid, Spain), and the variants were analyzed using “Data Genomics” software (v.2; Health in Code in Madrid, Spain). In silico pathogenicity prediction was analyzed with Alamut 2.7 (Sophia Genetics SA, Rolle, Switzerland) and other tools such as SIFT Ensembl 66 (SIFT scores range from 0 to 1. The smaller the score, the more likely the SNP has a damaging effect; damaging < 0.06); Polyphen-2 v2.2.2 (scores range from 0 to 1; benign < 0.03); Mutation Assessor, release 2 (scores range from −5.17 to 6.49 in dbNSFP; damaging > 1.8); FATHMM-MKL, v2.3 (Scores range from 0 to 1. SNVs with scores >0.5 are predicted to be deleterious, and those <0.5 are predicted to be neutral or benign. Scores close to 0 or 1 represent the highest confidence); GERP 2 version 2010 (scores range from −12.3 to 6.17, with 6.17 being the most conserved); PhyloP100way (Scores are based on multiple alignments of 99 vertebrate genome sequences to the human genome. The greater the score, the more conserved the site); CADD, v1.3 (scores above 20 are predicted to be among the 1.0% most deleterious possible substitutions in the human genome); DANN, v2014 (Scores range from 0 to 1. A larger number indicates a higher probability of being damaged). The allele frequency threshold used for variant filtering was 0.01 to avoid polymorphism higher than 1%. Population frequencies of the detected variants were assessed using the gnomAD exomes; gnomAD genomes; Bravo; Beacon; 1000 genome project; Spanish Exon Variant Project; and NHLBI exome sequencing project: ESP6500_EA_AF. Variants were finally classified according to the ACMG/AMP guidelines [23] according to the following characteristics: gene, variant, genetic inheritance, in silico predictors, bibliography, and clinical correlation. Additionally, this customized NGS panel is an ASD-specific panel; we consider three or more points in the ACMG classification as significant clinical criteria for the variant, thereby expecting the parental segregation analysis. Thus, a lower score, located in a range of 1–2 points, classified the variant as an uncertain type (VUS). A score of zero or negative classified the variant as benign.

### 2.2. Study Limitations

The main limitation of this study is the difficulty of finding individuals with primary autism who do not exhibit malformations or any form of dysmorphia when examined by clinical experts. We used the “presumptive diagnosis” to include patients in the study after they had visited our clinics. Such patients had mostly been referred by a neurologist. Systematic ASD diagnoses in our country are rather scarce, are often not diagnosed until later in life, and are sometimes revoked after treatment. In addition, the study’s findings may be influenced by the limited sample size and the absence of functional/segregation analysis regarding variants of uncertain significance (VUS). In addition, the absence of trios is another limitation. Without parental segregation studies, the clinical significance of the variants are, thus far, more difficult to elucidate.

## 3. Results

The final cohort consisted of 48 Spanish patients with ASD, with a median age of 8 years. Of these, 45 were males (93.75%) and 3 were females (6.23%). Within the cohort, 45 individuals (93.75%) were in the pediatric age range (<1 to 16 years).

Regarding the diagnoses, autism was observed in 34 patients (70.8%), nine cases of Asperger’s syndrome were observed (18.75%), and five cases of unspecified developmental disorders with ASD were observed (10.4%). In fact, in addition to ASD, other comorbidities were observed, such as intellectual disability (ID) and psychomotor delay (PMD) in 16 patients (33.3%), attention deficit hyperactivity disorder (ADHD) in 5 patients (10.4%), and epilepsy in 1 patient (2.8%).

Upon performing the NGS AutismSeq panel on the 48 patients, we found that 11 patients (23%) had variants classified as “pathogenic” (including pathogenic (P), probably pathogenic (LP), or VUS-likely pathogenic (VUS-LP)) (Table 1). Of these 11 patients with “pathogenic” variants, 100% were males, two had Asperger’s syndrome (18%), and only one had an intellectual disability (9%) as the presumptive diagnosis. Out of the 12 “pathogenic” variants found, there were 9 missense (75%), 2 frameshifts (16.66%), and 1 nonsense (8.33%). All variants were found in heterozygosity (Table 1).

A total of 10 patients (9 males (90%) and 1 female (10%)) had a total of 11 variants classified as variants of uncertain significance (VUS). They are distributed as follows: 10 were missense variations (90.9%) and 1 was an in-frame insertion variant (9.1%). All variants were also found in heterozygosity. From a diagnosis point of view; two of the patients had Asperger’s syndrome, one had PMD (psychomotor delay), one had ID, and another presented with epilepsy and ADHD (Attention Deficit Hyperactivity Disorder). The rest showed primary autism (Table 2). One of the variants was presented in a single individual with another “pathogenic” variation (see also Table 1).

**Figure 2:**
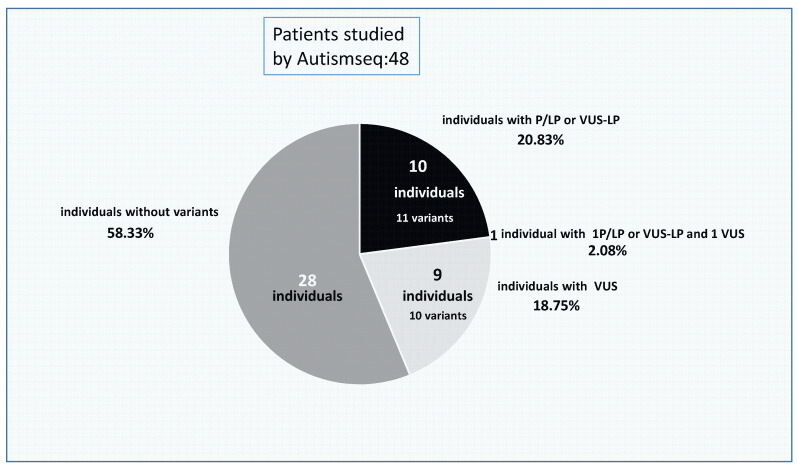
Distribution of the NGS panel results. Percentage of cases with “pathogenic” (P/LP or VUS-LP), VUS variants, and those patients without changes found by NGS. P/LP—pathogenic/probably pathogenic; VUS—variants of uncertain significance.

Finally, in 28 individuals (58.33%), no variants were found (Figure 2).

## 4. Discussion

Patients analyzed using AutismSeq mainly presented ASD, typically without other dysmorphias, malformations, or comorbidities. Consequently, they constituted a homogeneous group, which could be attributed to the fact that they had already undergone multiple molecular studies and clinical evaluations before reaching this study. However, it is necessary to note that many other patients with a higher degree of clinical diversity than that of ASD individuals have been already diagnosed. Thus, an important gap needs to be addressed. Here, we propose to analyze whether or not a putative implementation using a customized singleton panel for autism is a viable option, in a pilot study for our laboratory, which is a tertiary hospital in a public health system with no available economic refunds.

Besides possible limitations (see Section 2), we classified, with possible clinical relevance, several P/LP and VUS variants, considering likely pathogenic variants (VUS-LP) in 11 patients out of 48, which translates to an initial yield of around 23%. However this is not, a priori, a real diagnostic yield regarding ASD. Many of these P/LP or VUS-LP variants were not exhibited in relation to a diagnosis of autism. Some are related to autosomal recessive disorders, and in others, there is not enough solid evidence (although all selected genes in the panel were autism-related genes), or certain genes are associated with a different disorder unrelated to autism (e.g., case AUT99 with variants in *KCJN11* (diabetes) and *NBEAL1* (no disease association yet)). Thus, these results seem very similar to those found in a few other recent studies [20,24,25,26] using a gene panel and a very similar sample size, in our country (3–13%). The final diagnostic yield using this customized panel may be ranked in the higher part of the previous interval, probably between 10–14.5% (5–7 out of 11 previous individuals found). This fact could be attributed to a better selection of the genes included in the panel AutismSeq, (a joint effort between different experts in ASD and our clinical experience), and/or the cases selected. Without using trios or studying subsequent parental segregation of the SNVs, the findings are too speculative to extrapolate to a real yield; however, the findings can inform discussions regarding future approaches in our routine implementation for this group of patients.

Regarding the choice for ASD analysis between a gene panel and WES (whole exome sequencing) or WGS (whole genome sequencing), in terms of cost/analysis effort/diagnostic yield in our country, Codina-Solá and colleagues [27] obtained a diagnostic performance of 19% (7/36) using exome sequencing, which is a result similar to ours. This comparison suggests that our panel design approach has been quite successful both in terms of strategy and from an economic perspective and is suitable for the clinical setting. In our hands, the cost of one exome is around five times more expensive than a sample in a panel. In our public health system, WGS is still rarely used in clinical routines for economic reasons.

The fact that a customized gene panel has a similar diagnostic performance in some cases compared to WEs may, indeed, be influenced by the design, expertise in interpretation (due to its ease and the lower number of obtained SNVs in each sample), number of genes included, and of course, selection of patients. However, it is also important to note that systematic comparisons are difficult to establish, as these types of studies are infrequent due to economic constraints. Typically, panel-to-exome comparisons are not performed with the same cases, at least at this stage of the technology’s development. One of the main advantages of performing a customized gene panel in ASD patients compared to exome sequencing is that the customized gene panel is highly focused on the specific disease being studied—in this case, ASD. As a result, it ensures the sequencing of regions of interest exclusively, allowing for a greater depth of coverage compared to many situations with exome sequencing. In some cases, VUS that might go unnoticed in an exome analysis can be detected by a panel (personal data), as panel sequencing allows for the detection of low-frequency variants, yields less complex information compared to exome sequencing, and has a lower likelihood of unexpected findings. Exome sequencing, on the other hand, can be virtually “panelized” through bioinformatics approaches, although it such approaches do not improve the aforementioned limitation. However, exome sequencing reduces the limited design of NGS panels, where some less-frequent diagnoses or genes not included in the panel might be missed, especially if there is a recent association of a gene with ASD. Thus, exome sequencing allows for the correlation of new genes with new pathologies and phenotypes, although it comes with a higher economic cost. By contrast, the use of panels prevents the discovery of new genes associated with ASD. For all the above reasons, we decided to design a clinically useful panel for ASD (AutismSeq) to check whether or not a singleton customized NGS panel for ASD is cost-effective in our hands. Once validated and analyzed, the data obtained from this panel allowed us to decide whether or not to implement it for use in clinical routine settings for ASD analysis.

Regarding the detailed analysis of the identified SNVs, all “pathogenic” (P/PL, VUS-LP) variants and VUS were found in the heterozygous state; none were found in homozygosity or compound heterozygosity, and the majority of them were missense mutations. Interestingly, all results with pathogenic variants were observed in males (11/11; 100%), which is different from what we found in the previous analysis of CNVs (18/27; 67%) using CMA [22].

**Figure 3:**
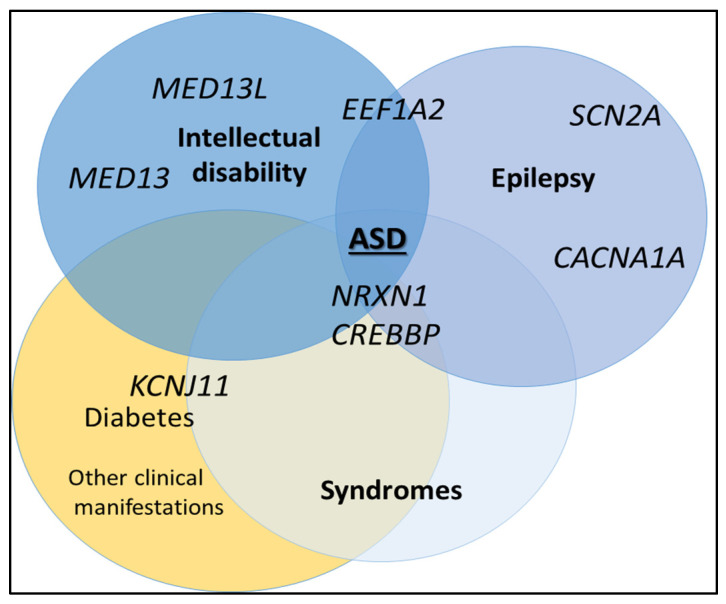
Groups of genes with pathogenic variations were detected using the NGS panel (AutismSeq) in their association with epilepsy, intellectual disability, or genetic syndromes.

Globally among all the variants found, we identified at least three groups of genes affected by these SNVs in the essential ASD cases: those related to epilepsy, those associated with intellectual disability, and those associated with more syndromic characteristics (Figure 3).

Regarding the first group, many of the gene changes have been previously associated with epilepsy, such as the *SCN2A* gene (AUT108 and AUT187). Interestingly, these patients have not shown epilepsy yet (this gene is associated with early onset epilepsy). This gene codes for a voltage-gated ion channel protein in nerves and muscles. Regarding ASD, this gene has been also described in association with developmental and epileptic encephalopathy 11 (OMIM #613721), episodic ataxia type 9 (OMIM #607745), and benign familial and infantile seizures (OMIM #607745), with an autosomal dominant inheritance mode. It has been independently identified previously as a strong candidate for ASD [28], and there are approximately 45 articles that support this association (source PUBMED; consulted June 2023). Another gene strongly associated with epilepsy is *CACNA1A*, for which we have found three different variants in three different patients (AUT117, AUT161, who also has an intellectual disability, and AUT221). One of them, we classified as VUS-LP, and the other two as VUS. *CACNA1A* is associated with hemiplegic migraine syndromes, although it has also been described in patients with ASD, intellectual disability, and even ADHD. There are several reports supporting this association [29,30,31]. This gene encodes for calcium channel subunits and is a transcription factor that regulates the expression of genes involved in nervous system development and Purkinje cell function. Despite its association with epilepsy, none of the patients in this cohort with these variants have developed epilepsy so far [29,32]. Finally, in the group of genes related to epilepsy, we found a variant in the *EEF1A2* gene that resulted in a VUS probably converted to LP change in the patient AUT149. Variants in this gene are associated with developmental and epileptic encephalopathy type 33 (OMIM #616409), intellectual disability type 38 (OMIM #616393), and ASD [33]. Again, this patient has not shown epilepsy so far (17 years old). *EEF1A2* encodes a protein responsible for the enzymatic delivery of aminoacyl-tRNA to the ribosome. The fact that many of these patients have pathogenic changes related to epilepsy but do not develop it (with autosomal dominant inheritance) suggests that these types of epilepsy could be the result of a multifactorial action, they may still yet develop it, or they could be influenced by other factors such as the incomplete penetrance phenomenon [34].

The SFARI database (https://sfari.org/; consulted 23 June 2023) provides significant evidence (>12 research articles) linking genes described earlier to primary autism. Thus, it is important to remark that some of the genes described earlier are associated with both primary and syndromic autism. Additionally, there are also variants in genes related to epilepsy, such as *CACNA1D* (AUT142), *CHD1* (AUT183), *KMT2E* (AUT184), and *CHD2* (AUT195). However, all these patients have primary autism without any comorbidities, meaning they do not currently present with epilepsy, making it difficult to attribute their clinical role in these ASD patients.

The other important group of “pathogenic” variants found were related to intellectual disability. The pathogenic variants in *MED13L* present in patient AUT23 are associated with impaired intellectual development and distinctive facial features with or without cardiac defects (OMIM #616789). At least 35 reports have linked this gene with ASD, reviewed by Iossifov et al., 2012 [35]. In addition, two de novo loss-of-function variants in *MED13L* have been identified in individuals with ASD from the Simons Simplex Collection database. The protein encoded by *MED13L* is a subunit of the mediator complex that functions as a transcriptional co-activator for genes transcribed by RNA polymerase II [36]. Our patient, in addition to ASD, presents mild intellectual disability without any cardiac problems. On the other hand, the *MED13* gene has been associated with intellectual developmental disorder 61 (OMIM #618009). Individuals with this disorder exhibit nonspecific facial features, significant language impairment, ASD, and ADHD, with varying phenotypes and severity. The *MED13* gene is considered a strong candidate for ASD itself [37]. Patient AUT118, who has a variant in the *MED13* gene, has been presumptively diagnosed with Asperger’s syndrome, but we classified the variant as VUS.

A third group of genes was associated with specific syndromes or pathologies. Patient AUT120 presented a pathogenic change in the *NRXN1* gene, which is related to Pitt–Hopkins-like syndrome (OMIM #614325), which we classified as VUS (limit, but with an autosomal recessive inheritance for this gen; Table 2). However, this syndrome was ruled out because our patient had a heterozygous variant, and only exhibited ASD, which was not compatible with the clinical presentation of the latest syndrome. However, this gene has been also proposed as part of a susceptibility region for schizophrenia (OMIM #614332), and the SFARI database shows a high degree of association with primary ASD, as evidenced by approximately 49 research articles. The *NRXN1* gene encodes neurexin, which is a cell adhesion molecule and cell surface receptor that binds to neuroligins at synapses in the central nervous system for proper neurotransmission [38].

Another well-known gene affected in one of the individuals in this cohort is *CREBBP*, whose pathogenic mutations are associated with Rubinstein–Taybi syndrome 1 (OMIM #180849) and Menke–Hennekam syndrome 1 (OMIM #618332), both inherited in an autosomal dominant manner. Although both syndromes include the condition of ASD, there is not enough clinical evidence to establish a diagnosis of either syndrome in our patient (AUT157) who has an LP variation in this gene. However, *CREBBP* has also been associated with primary autism in more than 10 research articles and the SFARI database, suggesting that it could explain the pure ASD clinical presentation in AUT157 [39]. The function of this gene is associated with the transcriptional co-activation of various transcription factors, as it binds to the cyclic adenosine monophosphate response element-binding protein (CREB). This gene plays critical roles in embryonic development, growth control, and homeostasis by coupling chromatin remodeling [40].

One of the genes frequently associated with VUS or VUS-LP in our study was the *CHD7* gene (found in three patients: AUT25, AUT114, and AUT115). Different pathogenic variants in this gene have been related to CHARGE syndrome (OMIM #214800) and hypogonadotropic hypogonadism type 5 with and without anosmia (OMIM #612370), both with a dominant inheritance pattern. However, none of these patients present other clinical characteristics apart from primary ASD.

Regarding VUS with a possible pathogenic cause, it is worth mentioning that patient AUT195 has a variant in the *SETD2* gene described in the literature regarding Luscan–Lumish syndrome (OMIM #616831), an overgrowth syndrome with which we have great clinical experience [41]. This variant could be compatible with the diagnosis, as the patient presents tall stature, hemihypertrophy, ASD, and DI. However, hemihypertrophy has not been previously described in association with this disorder [41].

Remarkably, we point out that, apparently, at the end, non-syndromic ASD cases show some association with regions previously involved in syndromic forms. This fact highlights the great variability and clinical and genetic heterogeneity of ASD cases. Most of the variants found are also related to intellectual disability (ID) and/or epilepsy, which suggests a possible interconnection between these different genes. It is also important to emphasize that numerous genetic syndromes exhibit incomplete penetrance and variable clinical expression, which can be observed both among individuals within the same family (intrafamilial variability) and among unrelated individuals (interfamilial variability). Establishing a genotype–phenotype correlation in this type of profile is very challenging. Environmental factors and/or coexisting genetic factors may modulate the expression of the genomic alteration in such a way that the phenotype is mild in one individual while causing a severe phenotype in another one.

Although our main objective was to be able to compare the situation in our country, trying to analyze the best situation when implementing the use of genomic technologies in our laboratory, it is interesting to compare our performance and those that are revealed in other international studies. In fact, in a recent study by Hu and colleagues (2023), the detection performance of a targeted and personalized panel in ASD patients showed a “P/LP” rate of 16.9% [42], which is quite in line with our performance and also other previous works (13.89%; 6.7–9.2%; 23.4% [43,44,45]; or 12%, combining FRAX, CMA, and NGS by panel [46]). Srivastava et al., 2019 [47] revealed a yield in the range of 30% to 40% for exome use in ASD patients and others, put it at 15.2% using Trios or 10.1% of families in a singleton manner [48]. Ni Ghralaigh et al., 2020 [49] showed a diagnostic yield in ASD of 31% with WES and 42.4% with WGS, with a high cost over a panel. Finally, a meta-analysis study revealed small differences between the use of a panel and WES in ASD families [50]. It is true that with the significant reduction in costs of massive parallel sequencing, these approaches, preferably through panels and perhaps soon with WES, may allow a greater number of ASD patients and others with neuro-alterations to benefit from genomic tests, whose positive results could give them more opportunities to access to new therapies.

## 5. Conclusions

A custom-designed panel of 311 genes (AutismSeq) was developed and validated. It could be a significant clinical tool in our hands for implementation into ASD analysis, although many factors have to be previously considered to allow for the implementation of the panel in routine clinical practice in our laboratory (e.g., regarding using trios, etc.). A major number of genes with pathogenic variants that are associated with epilepsy were identified through an NGS panel, despite the fact that none of the ASD patients currently present epilepsy. This may be due to incomplete penetrance, variable expressivity, or age of onset. Another group of genes affected by pathogenic variants includes those related to intellectual disability, such as *MED13L* and *MED13*, as well as others associated with certain syndromic conditions, such as *CREBBP* and *SETD2.* Consequently, we can establish the following candidate genes, which have been less frequently described concerning ASD: *MED13L*, *SCN2A*, *CACNA1A*, *MED13*, and *EEF1A2.*

**Figure 4:**
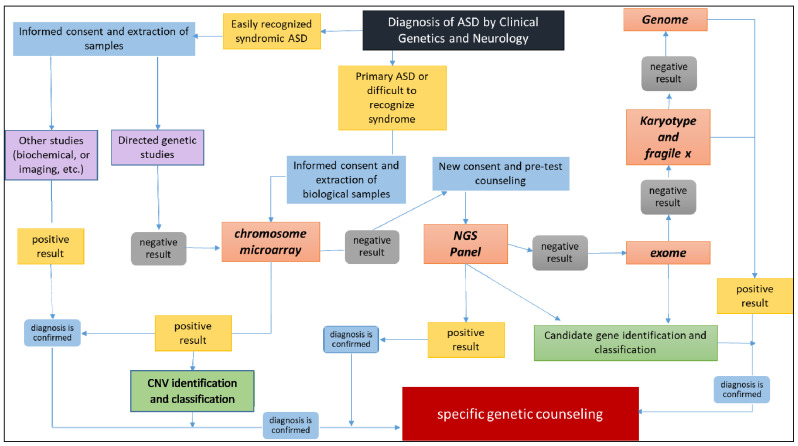
Analysis of the diagnostic algorithm for ASD using array-CGH (KaryoArray^®^)/NGS panel (AutismSeq).

Our results also support the significant genetic and clinical heterogeneity among individuals with ASD and the current challenges related to their molecular diagnosis. Therefore, we recommend the routine use of genomic techniques such as CMA [22] and NGS for ASD diagnosis. Insufficient utilization of these new genomic technologies in clinical practice has limited our understanding of the etiology of ASD. The use of both technologies, either as initial standalone studies or in combination, is critical. Based on our results, we propose the following diagnostic algorithm (Figure 4). We highlighted that there is one possibility that could be improved by including mitochondrial DNA (mtDNA) analysis and pharmacogenomics at some point of this scheme. On the other hand, many other laboratories worldwide may alter the positioning of the use of the genome versus Karyotyping and fragile X. This a matter of feasibility that requires consideration of the individual laboratories’ operational and economic limitations.

## Figures and Tables

**Table 1:** Pathogenic, probably pathogenic, and VUS-likely pathogenic variants found in the cohort of individuals with ASD by personalized NGS panel.

Patient/ Sex	Clinical Features in the Patient	Gen/Exon	cHGVS/ pHGVS/ Zygosity	Protein Effect dBSNP	OMIM (Gene Mainly Associated with)	Frequencies (gnomAD)	ACMG Classification	Conservation Predictors
AUT23 male	ASD	*MED13L* 5/31	NM_015335.5: c.572del (p.Leu191Ter) heterozygous	frameshift	# 616789 Impaired intellectual development and distinctive facial features with or without cardiac defects (AD)	Not Found	LP; PVS1, PM2 (9 pts; 9P-0B)	PhastCons100 Way: 1.00 PyloP100way: 5.488; 5.344 GERP RS: 4.72 median
AUT99 male	ASD	*KCNJ11* 1/1 *NBEAL1* 13/55	NM_0000525.4: c.325C>A p.(Pro109Thr) heterozygous NM_001114132.2: c.1741del p.Val581CfsTer20 heterozygous	missense rs758228551 frameshift	# 610582 Diabetes mellitus, transient neonatal 3 (AD) # 618856 Diabetes, permanent neonatal 2, with or without neurologic features (AD) # 601820 Hyperinsulinemic hypoglycemia, familial, 2 (AD y AR) # 616329 Maturity-onset diabetes of the young, type 13 (AD) none	Exomes: *f* = 0.00000398; not in Eur. NF Genomes: Not Found Not Found	VUS-LP; PM2, PP2, PP3 (3 pts; 3P-0B) PP3 pathogenic supporting: CADD, MutPred, MVP, PROVEAN, SIFT4G, DANN, MetaRNN, BayesDeladdAF VUS-LP; PVS1, PM2 (5 pts; 5P-0B)	PhastCons100 Way: 1.00 PyloP100way: 5.366 GERP RS: 3.99 PhastCons100 Way: 1.00 PyloP100way: 6.062 GERP RS: 5.519
AUT108 male	ASD	*SCN2A* 28/28	NM_001040142.2: c.5890G>A p.Asp1964Asn heterozygous	missense	# 613721 Developmental and epileptic encephalopathy 11 (AD) # 618924 Episodic ataxia, type 9 (AD) # 607745 Seizures, benign familial infantile, 3 (AD)	Exomes: *f* = 0.00000398; not in Eur. NF Genomes: Not Found	VUS-LP; PM2, PP2, PP3 (3 pts; 3P-0B) PP3 pathogenic supporting: CADD, EIGEN PC, MVP, PROVEAN, FATHAMM-MKL, DANN, MetaLR, MetaSVM	PhastCons100 Way: 1.00 PyloP100way: 8.017 GERP RS: 5.73
AUT114 male	ASD	*CHD7* 16/38	NM_017780.4: c.3973T>C p.Yyr1325His heterozygous	Missense rs377535841	# 214800 CHARGE syndrome (AD) # 612370 Hypogonadotropic hypogonadism 5 with or without anosmia (AD)	Exomes: *f* = 0.0000684 Genomes: *f* = 0.0000637	VUS-LP; PM1,PM5, PP3, BS2 (4 pts; 5P-1B) PP3 pathogenic supporting: CADD, EIGEN, EIGEN PC, LRT, LIST-S2, M_CAP, PROVEAN, SIFT DANN, MetaRNN, REVEL, BayesDel no AF, BayesDel addAF CliVar (conflicting VUS:5, LB:3); UNIPROT (LP); Varsome (VUS)	PhastCons100 Way: 1.00 PyloP100way: 8.042 GERP RS: 5.8
AUT115 male	ASD	*CHD7* 16/38	NM_017780.4: c.3973T>C p.Yyr1325His heterozygous	Missense rs377535841	# 214800 CHARGE syndrome (AD) # 612370 Hypogonadotropic hypogonadism 5 with or without anosmia (AD)	Exomes: *f* = 0.0000684 Genomes: *f* = 0.0000637	VUS-LP; PM1,PM5, PP3, BS2 (4 pts; 5P-1B) PP3 pathogenic supporting: CADD, EIGEN, EIGEN PC, LRT, LIST-S2, M_CAP, PROVEAN, SIFT DANN, MetaRNN, REVEL, BayesDel no AF, BayesDel addAF CliVar (conflicting VUS:5, LB:3); UNIPROT (LP); Varsome (VUS)	PhastCons100 Way: 1.00 PyloP100way: 8.042 GERP RS: 5.8
AUT117 male	ASD	*CACNA1A* 31/48	NM_001127221.2: c.4880G>A p.Arg1627His heterozygous	missense rs777769751	# 617106 Developmental and epileptic encephalopathy 42 (AD) # 108500 Episodic ataxia, type 2 (AD) # 141500 Migraine, familial hemiplegic, 1, with progressive cerebellar ataxia (AD) # 183086 Spinocerebellar ataxia 6 (AD)	Exomes: *f* = 0.0000443; in Eur. NF Genomes: Not Found	VUS-LP; PM2, PP3 (3 pts; 3P-0B) PP3 pathogenic supporting: CADD, M-CAP, PrimateAI MVP, PROVEAN, FATHAMM, DANN, MetaLR, MetaRNN, MetaSVN, REVEL; ClinVAR (VUS)	PhastCons100 Way: 1.00 PyloP100way: 4.918 GERP RS: 3.63
AUT142 male	ASD	*CACNA1D* 42/49	NM_001128840.3: c.4967G>A p.Arg1656His heterozygous	Missense rs890934509	# 615474 Primary aldosteronism, seizures, and neurologic abnormalities (AD) # 614896 Sinoatrial node dysfunction and deafness (AR)	Exomes: *f* = 0.00000398; in Eur. NF Genomes: not found	VUS-LP; PM2, PP3 (3 pts; 3P-0B) PP3 pathogenic supporting: CADD, EIGEN, EIGEN PC, PrimateAI, FATHAMM, FATHAMM-XF, M-CAP, LIST-S2, PROVEAN, FATHAMM-MKL, DANN, MetaLR, MetaRNN, MetaSVN, REVEL, BAyesDel no AF, BayesDel addAF	PhastCons100 Way: 0.996 PyloP100way: 2.496 GERP RS: 2.97
AUT149 male	Asperger depression	*EEF1A2* 4/8	NM_001958.5: c.479C>T p.Pro160Leu heterozygous	missense	# 616409 Developmental and epileptic encephalopathy 33 (AD) # 616393 Intellectual developmental disorder, autosomal dominant 38 (AD)	Not Found	VUS-LP; PM2, PP2, PP3 (3 pts; 3P-0B) PP3 pathogenic supporting: CADD, PrimateAI, LRT, PROVEAN, SIFT, FATHAMM-MKL, DANN, MetaRNN	PhastCons100 Way: 1.00 PyloP100way: 9.659 GERP RS: 3.866
AUT157 male	ASD	*CREBBP* 17/30	NM_004380.3: c.3559C>T p.Arg1187Ter heterozygous	nonsense	# 618332 Menke–Hennekam syndrome 1 (AD) # 180849 Rubinstein–Taybi syndrome 1 (AD)	Not Found	LP; PVS1, PM2 (9 pts; 9P-0B)	PhastCons100 Way: 1.00 PyloP100way: 7.389 GERP RS: 5.59
AUT 187 male	ASD	*SCN2A* 16/27	NM_001040142.2: c.2789A>C p.His930Pro heterozygous	missense	# 613721 Developmental and epileptic encephalopathy 11 (AD) # 618924 Episodic ataxia, type 9 (AD) # 607745 Seizures, benign familial infantile, 3 (AD)	Not Found	LP; PM1, PM2, PP3 (7 pts; 3P-0B) PP3 pathogenic supporting: CADD, EIGEN, EIGEN PC, DEOGEN2, MVP, PROVEAN, MVP, M-CAP, MutPred, FATHAMM, Mutation Assesor, PrimateAI, SIFT, SIFT4G FATHAMM-MKL, FATHAMM-XF, DANN, MetaLR, MetaSVM, REVEL, MetaRNN, BayesDel no AF, BayesDel Addai	PhastCons100 Way: 1.00 PyloP100way: 9.198 GERP RS: 5.42
AUT195 female	ASD ID Tall stature Hemi-hypertrophy	*SETD2* 15/21 *CHD2* 16/39	NM_014159.7: c.6299A>G p.Asp2100Gly heterozygous NM_001271.4: c.1994C>T p.Pro665Leu heterozygous	Missense Missense	# 616831 Luscan–Lumish syndrome (AD) # 615369 Developmental and epileptic encephalopathy 94 (AD)	Exomes: *f* = 0.00000401, in Eur. NF Genomes: not found Exomes: *f* = 0.0000559, in Eur. NF Genomes: not found	VUS; PM2, BP1 (1 pts; 2P-1B) PP3 pathogenic supporting: CADD, DANN, FATHAMM-MKL LP; PM1, PM2, PP3, (7pts; 6P-0B) PP3 pathogenic supporting: CADD, DANN, DEOGEN2, EIGEN, EIGEN PC, MVP, PROVEAN, FATHAMM_MKL, M-CAP, PrimateAI, SIFT, SIFT4G, METARNN, BayesDel addAF, BayesDel no AF, MetaLR, MetaSVM, REVEL, ClinVar (conflicting)	PhastCons100 Way: 1.00 PyloP100way: 6.81 GERP RS: 4.84 PhastCons100 Way: 1.00 PyloP100way: 7.817 GERP RS: 5.82

ASD—autism spectrum disorder; AD—autosomal dominant; AR—autosomal recessive and ID—intellectual disability.

**Table 2:** VUS found in the cohort of individuals with ASD by personalized NGS panel.

Patient/ Sex	Clinical Features in the Patient	Gen/Exon	cHGVS/ pHGVS/ Zygosity	Protein Effect dBSNP	OMIM (Gene Associated with.)	Frequencies (gnomAD)	ACMG Classification	Conservation Predictors
AUT118 male	Asperger	*MED13* 2/30	NM_005121.3: c.124C>T (p.Pro42Ser)/ heterozygous	missense rs778909357	# 618009 Intellectual developmental disorder, autosomal dominant 61 (AD)	Exomes: *f* = 0.0000579; not in Eur.NF Genomes: Not Found	VUS; PM2, PP3 (2 pts; 2P-0B) PP3 pathogenic supporting: CADD, EIGEN, EIGEN PC, LRT, PrimateAI, MVP, PROVEAN, FATHAMM-XF, DANN, MetaRNN	PhastCons100 Way: 1.00 PyloP100way: 9.873 GERP RS: 5.67
AUT120 male	ASD	*NRXN1* 1/7	NM_004801.5: c.77_79dup p.Gly26dup heterozygous	In-frame Insertion rs766368745	#614325 Pitt–Hopkins-like syndrome 2 (AR)	Not Found	VUS; PM4, PM2 (3 pts; 3P-0B) No info	PhastCons100 Way: 1.00 PyloP100way: 4.046, 5.708, 1, 43, 3.125 GERP RS: 5.05 median
AUT140 male	ASD ADHD epilepsy	*CEP290* 19/54	c.1834C>T p.Leu612Phe heterozygous	Missense	615991 Bardet–Biedl syndrome 14 (AR) # 610188 Joubert syndrome 5 (AR) # 611755 Leber congenital amaurosis 10 # 611134 Meckel syndrome 4 (AR) # 610189 Senior–Loken syndrome 6 (AR)	Not Found	VUS; PM2, PP3 (2 pts; 2P-1B) PP3 pathogenic supporting: CADD, EIGEN, EIGEN PC, FATHAMM-MKL, DANN	PhastCons100 Way: 1.00 PyloP100way: 7.562 GERP RS: 5.349
AUT147 male	ASD	*TCF20* 2/6	NM:_001378418.1: c.454T>G p.Tyr152Asp heterozygous	Missense	# 618430 Developmental delay with variable intellectual impairment and behavioral abnormalities (AD)	Not Found	VUS; PM2, PP3 (1 pts; 2P-1B) PP3 pathogenic supporting: CADD, EIGEN, EIGEN PC, PrimateAI, SIFT, SIFT4G, FATHAMM-MKL, DANN, BAyesDel no AF, BayesDel addAF	PhastCons100 Way: 1.00 PyloP100way: 8.785 GERP RS: 5.519
AUT152 male	ASD PMD	*SPTBN1* 25/36 *ZEB2* 7/9	NM_003128.3: c.5014C>T p.Arg1672Trp heterozygous NM_014795.4: c.1769T>C p.Leu590Pro heterozygous	Missense rs755243358 Missense	# 619475 Developmental delay, impaired speech, and behavioral abnormalities (AD) # 235730 Mowat–Wilson syndrome (AD)	Exomes: *f* = 0.0000161 Genomes: *f* = 0.0000319 Not Found	VUS; PM2, PP3 (2 pts; 2P-0B) PP3 pathogenic supporting: CADD, EIGEN, EIGEN PC, PrimateAI, SIFT, SIFT4G, FATHAMM-MKL, DANN, BAyesDel no AF, BayesDel addAF VUS; PM2, PP3, BP1 (1 pts; 2P-1B) PP3 pathogenic supporting: CADD, EIGEN, EIGEN PC, PrimateAI, LRT, MCAP, MutPred, LIST-S2 FATHAMM-MKL, DANN, BAyesDel no AF, BayesDel addAF REVEL, MetaRNN	PhastCons100 Way: 1.00 PyloP100way: 8.785 GERP RS: 5.519 PhastCons100 Way: 1.00 PyloP100way: 9.339 GERP RS: 5.75
AUT161 male	ASD ID	*CACNA1A* 46/48	NM_023035.3: c.6512G>A p.Arg2171His heterozygous	missense rs727503832	# 617106 Developmental and epileptic encephalopathy 42 (AD) # 108500 Episodic ataxia, type 2 (AD) # 141500 Migraine, familial hemiplegic, 1, with progressive cerebellar ataxia (AD) # 183086 Spinocerebellar ataxia 6 (AD)	Exomes: *f* = 0.000326; in Eur.NF Genomes: *f* = 0.000195; in Eur.NF	VUS; PM2, PP3 (2 pts; 2P-0B) PP3 pathogenic supporting: CADD, LIST-S2, PrimateAI, M-CAP, FATHAMM, DANN, MetaLR, BayesDel addAF ClinVAR (conflicting, 1star)	PhastCons100 Way: 1.00 PyloP100way: 5.001 GERP RS: 3.38
AUT171 male	ASD	*KMT2D*	NM_003482.4: c.13885A>C p.Thr4629Pro heterozygous	Missense	# 147920 Kabuki Syndrome 1	Not Found	VUS; PM2, PP3, BP1 (1 pts; 2P-1B) PP3 pathogenic supporting: CADD, EIGEN, EIGEN PC, PrimateAI, FATHAMM-XF, MCAP, MutPred, PROVEAN FATHAMM-MKL, DANN, AF, BayesDel addAF; LOVD(VUS)	PhastCons100 Way: 1.00 PyloP100way: 7.972 GERP RS: 5.579
AUT183 male	Asperger	*CHD1* 3/35	(NM_001270.4): c.315G>C p.Gln105His heterozygous	Missense rs906013276	# 617682 Pilarowski–Bjornsson syndrome (AD)	Not Found	VUS; PM2, PP2, (2 pts; 2P-0B) PP3 pathogenic supporting: CADD, DANN, many others uncertain	PhastCons100 Way:1.00 PyloP100way: 1.34 GERP RS: 4.789
AUT 221 male	ASD	*CACNA1A* 1/48	NM_023035.3: c.115G>A p.G39S p.Gly39Ser heterozygous	missense	# 617106 Developmental and epileptic encephalopathy 42 (AD) # 108500 Episodic ataxia, type 2 (AD) # 141500 Migraine, familial hemiplegic, 1, with progressive cerebellar ataxia (AD) # 183086 Spinocerebellar ataxia 6 (AD)	Not Found	VUS; PM2, PP3 (2 pts; 2P-0B) PP3 pathogenic supporting: CADD, PrimateAI, M-CAP, FATHAMM, DANN, MetaLR	PhastCons100 Way: 0.996 PyloP100way: 2.496 GERP RS: 2.97

ASD—autism spectrum disorder; PMD—psychomotor delay; ADHD—Attention Deficit Hyperactivity Disorder; ID—intellectual disability; AD—autosomal dominant; AR—autosomal recessive.

## Data Availability

Data are contained within the article and Supplementary Materials.

## Supplementary Materials

The following supporting information can be downloaded at: https://www.mdpi.com/article/10.3390/genes14112091/s1, Table S1: List of the 311 genes selected and included in the AutismSeq NGS custom panel; Table S2: Cases with variants used in the validation of the AutismSeq panel.
