# Supplementary material for: NGS Custom Panel Implementation in Patients with Non-Syndromic Autism Spectrum Disorders in the Clinical Routine of a Tertiary Hospital
Source: Genes (Basel). 2023 Nov 17;14(11):2091. doi: 10.3390/genes14112091 (PMC10671584; doi:10.3390/genes14112091)
Supplement: Supplementary file 1 [file genes-14-02091-s001.zip › genes-2581930-supplementary/Supplemental.pdf]

Table S1. Supplemental data. List of the 311 genes selected and included in the AutismSeq NGS custom panel.

ACSL4	*ADNP*	*ADSL*	*AFF2*	*AHDC1*	*AHI1*	*ALDH5A1*	*ANK2*	*ANK3*
ANKRD11	*ARHGEF9*	*ARID1B*	*ARX*	*ASH1L*	*ASTN2*	*ASXL3*	*ATP2B2*	*ATRX*
AUTS2	*BAZ2B*	*BCKDK*	*BCL11A*	*BDNF*	*BPTF*	*BRAF*	*BRPF1*	*BRWD3*
C12orf57	*CACNA1A*	*CACNA1B*	*CACNA1C*	*CACNA1D*	*CACNA1E*	*CACNA2D3*	*CADPS2*	*CAMK2A*
CAMK2B	*CDKL5*	*CELF4*	*CEP290*	*CHD1*	*CHD2*	*CHD3*	*CHD5*	*CHD7*
CHD8	*CHRNA4*	*CHRNA7*	*CIC*	*CLCN4*	*CMIP*	*CNKSR2*	*CNOT3*	*CNTN4*
CNTNAP2	*COL4A3BP*	*CREBBP*	*CTCF*	*CTNNB1*	*CUL3*	*CUX2*	*CYFIP1*	*DDX3X*
DEAF1	*DEPDC5*	*DHCR7*	*DHX30*	*DISC1*	*DLG4*	*DLGAP2*	*DMD*	*DMPK*
DNM1	*DNMT3A*	*DPP10*	*DPYD*	*DPYSL2*	*DSCAM*	*DYRK1A*	*EEF1A2*	*EHMT1*
EIF4E	*ELAVL3*	*ELN*	*EP300*	*EP400*	*EPHB2*	*FAT1*	*FBXO11*	*FGD1*
FMR1	*FOLR1*	*FOXG1*	*FOXP1*	*FOXP2*	*FRYL*	*GABRA4*	*GABRB2*	*GABRB3*
GAMT	*GIGYF1*	*GLRA2*	*GNAI1*	*GNB1*	*GRIA1*	*GRIA2*	*GRIA3*	*GRIN1*
GRIN2A	*GRIN2B*	*GRIP1*	*GRM5*	*HCN1*	*HDAC8*	*HDLBP*	*HECTD4*	*HECW2*
HEPACAM	*HIRA*	*HIVEP2*	*HNRNPH2*	*HNRNPU*	*HOMER1*	*HOXA1*	*HRAS*	*IQSEC2*
IRF2BPL	*ITPR1*	*JMJD1C*	*KANSL1*	*KAT6A*	*KCNB1*	*KCND2*	*KCNJ10*	*KCNJ11*
KCNQ2	*KCNQ3*	*KDM5C*	*KDM6A*	*KDM6B*	*KIAA0232*	*KIF5C*	*KMT2A*	*KMT2C*
KMT2D	*KMT2E*	*KMT5B*	*KRAS*	*LDB1*	*LRP1*	*LRRC4C*	*MAGEL2*	*MAOA*
MAP2	*MAP2K1*	*MAPK3*	*MAPT*	*MBD5*	*MDGA2*	*MECP2*	*MED12*	*MED13*
MED13L	*MEF2C*	*MTF1*	*MTOR*	*MUC5B*	*MYO9B*	*MYT1L*	*NAA15*	*NACC1*
NBEA	*NCOA1*	*NEXMIF*	*NF1*	*NFIA*	*NFIX*	*NIPBL*	*NLGN2*	*NLGN3*
NLGN4X	*NR2F1*	*NR4A2*	*NRXN1*	*NRXN2*	*NRXN3*	*NSD1*	*NTNG1*	*NTRK2*
OPHN1	*PACS1*	*PACS2*	*PAFAH1B1*	*PAK2*	*DLG1*	*PAX5*	*PAX6*	*PCDH10*
PCDH19	*PCDH9*	*PDE4A*	*PEX7*	*PHF12*	*PHF2*	*PHF21A*	*PHIP*	*PIK3R2*
POGZ	*PPM1D*	*PPP2R1A*	*PPP2R5D*	*PPP3CA*	*PPP5C*	*PQBP1*	*PRR12*	*PTCHD1*
PTEN	*PTK7*	*PTPN11*	*QRICH1*	*RAB39B*	*RAI1*	*RALGAPA1*	*RALGAPB*	*RAPGEF4*
RBFOX1	*RELN*	*RERE*	*RFWD2*	*RFX3*	*RIMS3*	*RORA*	*RORB*	*RPL10*
SATB2	*SCN1A*	*SCN2A*	*SCN8A*	*SET*	*SETBP1*	*SETD2*	*SETD5*	*SHANK1*
SHANK2	*SHANK3*	*SIK1*	*SIN3A*	*SLC6A1*	*SLC6A8*	*SLC7A5*	*SLC9A6*	*SMAD4*
SMARCA2	*SMARCA4*	*SMARCC2*	*SMC1A*	*SMC3*	*SND1*	*SNRPN*	*SON*	*SOX11*
SOX5	*SPAST*	*SPEN*	*SPRED1*	*SPTAN1*	*SPTBN1*	*SRPR*	*STAG1*	*STXBP1*
SYN1	*SYNCRIP*	*SYNGAP1*	*TAF1*	*TBL1XR1*	*TBR1*	*TCF20*	*TCF4*	*TCF7L2*
TLK2	*TM9SF4*	*TMLHE*	*TNRC6B*	*TRAF7*	*TRIO*	*TRIP12*	*TRRAP*	*TSC1*
TSC2	*UBE3A*	*UBE3C*	*UBR4*	*UPF3B*	*USP9X*	*VEZF1*	*VPS13B*	*WAC*
WASF1	*WDFY3*	*WDR26*	*WDR45*	*WNK3*	*YWHAG*	*YY1*	*ZBTB18*	*ZBTB20*
ZEB2	*ZMYND11*	*ZMYND8*	*ZNF292*	*ZNF462*				

Table S2 Supplemental data. Cases with variants used in the validation of the AutismSeq panel

**NM_001042492.3(*NF1*):c.2T>G (p.Met1Arg)**	Síndrome Neurofibromatosis 1 (OMIM #162200)
**NM_001374258.1(*BRAF*):c.1712G>C (p.Trp571Ser)**	Síndrome Noonan 7 (OMIM #613706)
**NM_004380.3(*CREBBP*):c.5905C>T (p.Gln1969Ter)**	Síndrome Rubinstein-Taybi (OMIM #180849)
**NM_022455.5(*NSD1*):c.1262G>A (p.Trp421Ter)**	Síndrome Sotos (OMIM #117550)
**NM_022552.5(*DNMT3A*):c.2645G>T (p.Arg882Leu)**	Síndrome Tatton-Brown-Rahman (OMIM #615879)
**NM_014141.6(*CNTNAP2*):c.97+1G>A**	Síndrome Pitt-Hopkins-like (OMIM #610042)
**NM_001372044.2(*SHANK3*):c.574A>T (p.Lys192Ter)**	Síndrome Phelan-McDermid (OMIM #606232)
**NM_130839.5(*UBE3A*):c.2618A>T (p.Ter873Leu)**	Síndrome Angelman (OMIM #105830)
**NM_014795.4(*ZEB2*):c.3211T>C (p.Ser1071Pro)**	Síndrome Mowat-Wilson (OMIM #235730)
**NM_152594.3(*SPRED1*):c.148C>T (p.Gln50Ter)**	Síndrome Legius (OMIM #611431)
